# Target Identification and Intervention Strategies against Kinetoplastid Protozoan Parasites

**DOI:** 10.4061/2011/185413

**Published:** 2011-08-09

**Authors:** Hemanta K. Majumder, Wanderley de Souza, Kwang Poo Chang

**Affiliations:** ^1^Division of Infectious Diseases and Immunology, Indian Institute of Chemical Biology, 4 Raja S. C. Mullick Road, Jadavpur, Kolkata 700 032, India; ^2^Instituto de Biofisica Carlos Chagas Filho, UFRJ Directoria de Programas do Inmetro, Rio de Janeiro 21941-902, Brazil; ^3^Chicago Medical School, Rosalind Franklin University of Medicine and Science, North Chicago, IL 60064, USA

The past few decades have been marked by numerous admirable research efforts and promising technological advancements in the field of research on protozoan parasites. The parasites of this genre cause some devastating diseases that pose alarming threat to the mankind. Though several intervention strategies have been developed to get rid of these parasites, they always seem to frustrate the efforts of the scientific community sooner or later. The intervention strategies include identification of novel drug targets, development of target-based therapy, and development of vaccines. that provide significant impetus in the field of research pertaining to these parasites. In this context, several reviews have appeared in the past few years elucidating different drug targets in these parasites. For example, Das et al. [1], Balaña-Fouce et al. [2], and others have described the role of topoisomerases as potential drug targets in these kinetoplastid protozoa. Urbina [3] has described the lipid biosynthetic pathway as a possible chemotherapeutic target whereas McConville [4] has elucidated the potential of parasite surface glycoconjugates as possible drug targets. Other targets include cysteine peptidases [5] and histone deacetylases [6] of the trypanosomatid parasites.

Parasites of the genus *Trypanosoma* and *Leishmania* are kinetoplastid protozoan parasites that cause trypanosomiasis and leishmaniasis, respectively. Parasites belonging to the genus *Plasmodium* mainly cause malaria. These diseases are prevalent in tropical and subtropical countries and cause significant morbidity and mortality. However, these diseases are of the lowest priority because they offer little or no commercial incentives to the pharmaceutical companies.

This special issue is a much needed and timely compilation of selected research and review articles in the concerned field. Though the selected papers are not a comprehensive representation of the field, but they represent a rich mixture of multifaceted knowledge that we have the pleasure of sharing with the readers. We would like to thank all the authors for their excellent contributions and also the reviewers for their efforts in assisting us.

This special issue contains thirteen papers, of which five are research papers, and the rest are review articles. The five research articles mainly focus on development of new drugs and targets and also shed light on novel therapeutic intervention strategies. In the first paper, S. Sengupta et al. have established cryptolepine-induced cell death in the protozoan parasite *L. donovani*. Interestingly, the death process is augmented when the autophagic mechanism is inhibited by specific chemical inhibitors, and this finding may form the skeleton for novel therapeutic intervention strategies. 

In the second paper, S. Teixeira de Macedo-Silva et al. have investigated the effect of the antiarrhythmic drug amiodarone on the promastigotes and amastigotes form of *L. amazonensis*. They have shown that this drug has antiproliferative effect on *L. amazonensis* promastigotes and amastigotes and causes depolarization of mitochondrial membrane potential in both forms which ultimately leads to cell death of the parasites. So this compound may serve as a potential starting material for antileishmanial drug development.

In the third paper, L. Major and T. K. Smith. have screened the MayBridge Rule of 3 Fragment Library to identify compounds targeting Inositol-3-phosphate synthase (INO1) which has previously been genetically validated as a drug target against *Trypanosoma brucei*, the causative agent of African sleeping sickness. By this approach, they have identified 38 compounds that significantly altered the Tm of TbINO1. Four compounds showed trypanocidal activity with ED50s in the tens of micromolar range, with 2 having a selectivity index in excess of 250.

Topoisomerases are key enzymes that play a pivotal role in various cellular processes and also serve as an important drug target. In the paper, A. Roy et al. have described a synthetic peptide, WRWYCRCK, with inhibitory effect on the essential enzyme topoisomerase I from the malaria-causing parasite *Plasmodium falciparum.* Although *Plasmodium falciparum* does not belong to the order kinetoplastida, but still it has several features common with the kinetoplastid protozoan parasite *T. brucei*, for example, antigenic variation. The transition step from noncovalent to covalent DNA binding of *P. falciparum *topoisomerase I is specifically inhibited by this peptide while the ligation step of catalysis remains unaffected. Molecular docking analyses further provide a mechanistic explanation for this inhibition. This work provides evidence that synthetic peptides may represent a new class of potential antiprotozoan drugs.

In the fifth paper, J. Kaur et al. have performed bioinformatic analysis of the *Leishmania donovani *long-chain fatty acid Co-A ligase (LCFA) as a novel drug target. The authors have previously found this enzyme to be differentially expressed by *Leishmania donovani *amastigotes resistant to antimonial treatment. In the present study, the authors have confirmed the presence of long-chain fatty acyl CoA ligase gene in the genome of clinical isolates of *Leishmania donovani *collected from the disease-endemic area in India and propose that this enzyme serves as an important protein and a potential target candidate for development of selective inhibitors against leishmaniasis.

This special issue also features some timely and much needed review articles in the field. In the sixth paper, S. Gupta et al. have validated the role of a key enzyme, glucose-6-phosphate dehydrogenase (G6PDH) in trypanosomatids as an important drug target and discussed the possibility of drug discovery targeting this enzyme. G6PDH is the first enzyme of the pentose phosphate pathway and is essential for the defense of the parasite against oxidative stress. *T. brucei *and *T. cruzi *G6PDHs are inhibited by steroids such as dehydroepiandrosterone and derivatives in an uncompetitive way. The *Trypanosoma *enzymes are more susceptible to inhibition by these compounds than the human G6PDH. These compounds are presently considered as promising leads for the development of new parasite-selective chemotherapeutic agents.

In the seventh paper, A. F. Coley et al. have discussed the possibility of therapeutic development targeting glycolysis in African trypanosomes. The parasite is limited to using glycolysis of host sugar for ATP production while infecting the human host. This dependence on glucose breakdown presents a series of targets for potential therapeutic development, many of which have been explored and validated as therapeutic targets experimentally and has been addressed in this paper in detail.

In the eighth paper, S. L. de Castro et al. have given a good overview of experimental chemotherapy in the Chagas disease which is caused by *Trypanosoma cruzi,* and it affects approximately eight million individuals in Latin America. The authors have presented a nice biochemical and proteomic overview of potential *T. cruzi* targets with reference to amidine derivatives and naphthoquinones that have showed the most promising efficacy against *T. cruzi*.

In the ninth paper, A. K. Haldar et al. have classically demonstrated the current status and future directions for the use of antimony in the treatment of leishmaniasis. The standard treatment of Kala-azar in the recent past has been the use of pentavalent antimonials (SbV) but there has been progressive rise in treatment failure to Sb(V) due to the problem of chemoresistance that has limited its use in the treatment program in the Indian subcontinent. However, it has been shown recently that some of the peroxovanadium compounds have Sb(V) resistance modifying ability in experimental infection with Sb(V) resistant *Leishmania donovani *isolates in murine model. Thus vanadium compounds may be used in combination with Sb(V) in the treatment of Sb(V) resistance cases of kala-azar.

In the tenth paper, R. Duncan et al. have presented a comprehensive overview of the genes involved in *Leishmania *pathogenesis with reference to the potential for drug target selection. Proteins that are differentially expressed or required in the amastigote life cycle stage found in the patient are likely to be effective drug targets. Several examples and their potential for chemotherapeutic disruption have been presented in this paper. The programmed cell death pathway that is now recognized among protozoan parasites is reviewed for some of its components and evidence that suggests that they could be targeted for anti-parasitic drug therapy has been presented.

In the next paper, A. Biswas et al. have discussed the role of cAMP signaling in the survival and infectivity of the protozoan parasite *Leishmania donovani*. While invading macrophages,* L. donovani* encounters striking shift in temperature and pH that act as the key environmental trigger for differentiation and increase cAMP level and cAMP-mediated responses. A differentially expressed soluble cytosolic cAMP phosphodiesterase (LdPDEA) might be related to infection establishment by shifting trypanothione pool utilization bias toward antioxidant defense. This paper explains the significance of cAMP signaling in parasite survival and infectivity.

In the twelfth paper, Md. Shadab and N. Ali have elegantly discussed the evasion of host defense mechanism by *L. donovani*. They have presented a detailed account of the subversion and signaling pathways that allow the parasites to get rid of the host defense mechanism.

In the last paper, A. Ghoshal and C. Mandal have presented a detailed perspective of sialic acids that serve as important determinants influencing the parasite biology. Despite the steady progress in the field of parasite glycobiology, sialobiology has been a less traversed domain of research in leishmaniasis. This paper focuses on identification, characterization, and differential distribution of sialoglycotope having the linkage-specific 9-*O*-acetylated sialic acid in promastigotes of different *Leishmania sp. *causing different clinical ramifications.

There are other areas of relevance not covered in the volume, that is, prophylactic and therapeutic vaccination, targeted drug delivery, and antigenic variation. However, the present issue covers a significant area of the subject and will be of immense interest to the readers.

*Hemanta K. Majumder* *Hemanta K. Majumder* *Wanderley de Souza* *Wanderley de Souza* *Kwang Poo Chang* *Kwang Poo Chang*
